# Strength, Deformation, and Acoustic Emission Characteristics of Raw Coal and Briquette Coal Samples under a Triaxial Compression Experiment

**DOI:** 10.1021/acsomega.1c03543

**Published:** 2021-11-16

**Authors:** Han Meng, Yuzhong Yang, Liyun Wu

**Affiliations:** School of Energy Science and Engineering, Henan Polytechnic University, Jiaozuo 454003, China

## Abstract

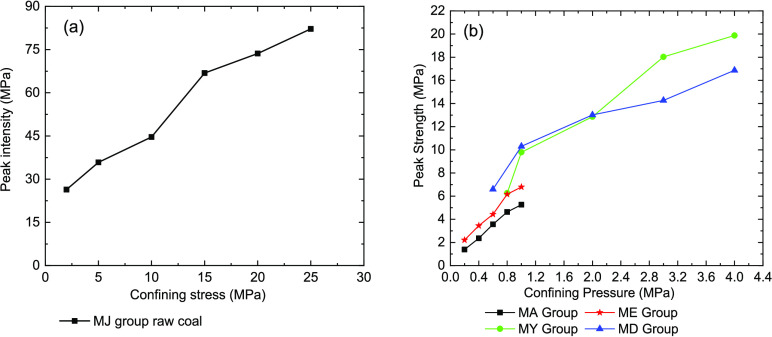

Raw coal and briquette coal samples have similar deformation characteristics. Addition of binders added into briquette coal could change the coal property. To better capture the characteristics of briquette coal samples in comparison to raw coal samples, we performed triaxial compression tests on raw coal and briquette coal samples with 7% content of four different binders. The experiment results show that the MD group (7% rosin) briquette coal has strong similarities to raw coal samples in strength, deformation, and acoustic emission (AE) features. We find that although four different binders (water, cement, rosin, and coal tar) are added into the briquette coal samples, the failure characteristic has high consistence. Briquette coal samples always show plastic failure, but raw coal always shows brittle failure. The increase in raw and briquette coal samples’ peak strength is associated with an increase in the confining pressure constant. However, as the confining pressure constant increases, the raw and briquette coal samples’ residual strength gradually reaches close to the peak strength. After the peak strength is reached, briquette coal samples always show a stronger strain and the raw coal samples have a weaker strain characteristic. AE events have a peak value on compression and elastic stage of briquette coal samples. AE events do not show a positive correlation relationship with the material strength of the briquette coal samples. Our study highlights that briquette coal samples with 7% rosin have more similarity in strength, deformation, and AE characteristic with raw coal samples.

## 1 Introduction

China has the largest number of coal mines and is the biggest coal producer in the world. Coal as a significant fossil energy source occupies a major position in China’s energy consumption structures. With the depletion of shallow resources, deep mining has become a common choice for the constant development of coal mines.^1,2^ In the Pingdingshan mining area, most of the mines have reached the depth of 800 m below sea level. Under the conditions of deep mining, the ground stress, gas pressure, and ground temperature all show obvious increase in characteristics.^3,4^ Hence, the deep mining conditions are extremely complicated. To realize safe and highly efficient mining in deep mines, a comprehensive research on the failure features of coal and rock masses is necessary.

Coal is essentially a solid with a well-developed pore system. The pore structure of coal plays a major role in its ability to adsorb methane, permeability characteristics, strength of coal.^5−7^ In most conditions, even coal samples taken from the same working face may show different properties. This indicated that the coal samples have high heterogeneity. In the Pingdingshan mining area, most of the mine’s coal seams were relatively soft; therefore, it was difficult to make standard raw coal samples. Even if some raw coal samples can be successfully prepared, these coal samples cannot represent the geological conditions of all coal mining areas in the mine. Therefore, using briquette coal to simulate raw coal has practical significance.

Raw coal is transformed from the remains of plants through a very long and complicated process of biochemistry, physical chemistry, and geochemistry. Briquette coal samples are manmade; they consistent of coal powder and different kinds of binders, and are then manufactured in a mold under a pressure machine. Some literature studies have shown that raw and briquette coal samples have certain similarities in physical characteristics.^8,9^ Acoustic emission (AE) technology is often used to monitor the changing process of internal fracture in coal and rock masses.^10−13^ When the rock material mass is deformed under loading, certain AE events will appear. Raw and briquette coal samples also show certain AE events, by studying which we can find their similar characteristics.

In numerous research studies, the mechanical parameters of the coal rock of triaxial experiments have shown that the process can be divided into several typical failure stages.^14−17^ These laboratory research studies have also shown the relationship between the failure and strength characteristics of coal rock with the confining pressure.^18^ AE is the vibration phenomenon that occurs during the destruction of coal and rock masses; the characteristics of cracks can be summarized as follows.^19−26^ The rock failure process is mainly controlled by crack propagation and fractional sliding with the cracks, through the AE monitoring system.^27,28^ More recently, AE technology has been adopted to divide Beishan granite cracks into elastic, failure, and residual stages.^29^ The plastic characteristics and microcracks of the deep coal body were more obvious, and the AE signals would be advanced when those are destroyed during compression.^30,31^

Most of the relevant literature are about the uniaxial, triaxial, and AE experiments of coal and rock samples, but there are few relevant studies about the different binders of briquette coal samples. The deformation and strength of raw and briquette coal have a certain consistency, and briquette coal samples were shown to exhibit plastic deformation characteristics.^32,33^ The uniaxial compression strength and elastic modulus of the reconstituted coal samples were similar to those of raw coal samples.^34−37^ The porosity of briquette coal samples is different, and the uniaxial compression strength and the adsorbed gas characteristics are also different.^38^ Our study aims to find one type of binder briquette coal samples that are similar to the raw coal samples under triaxial compression. We compared the deformation, strength, and AE event features of raw and briquette coal samples. The mechanical parameters are always influenced by the confining pressure; different binders have different influences on the failure parameters of briquette coal samples. This contribution by the way of an experimental study in which we perform a constant triaxial compression on raw and briquette coal samples can promote the search for a suitable material to simulate raw coal.

## 2 Coal Samples and Experimental Methods

### 2.1 Coal Samples and Preparation

The big-blocks raw coal were taken from 11110 coal mining face in no. 13 coal mines of the Pingdingshan Tian’an coal group. The surface of the block coal was covered with a plastic wrap and then transported into the laboratory. This coal is a typical coking coal with a high calorific value and an ultralow sulfur content. The apparent density of the coal is 1.43 t/m^3^. The microscopic composition of raw coal quantitatively shows that the ash content is 15.75%, total sulfur is 0.46%, carbon is 90.42%, hydrogen is 4.64%, phosphorus is 0.021% on average, and arsenic is on average 2.15 ppm.

**Figure 1:**
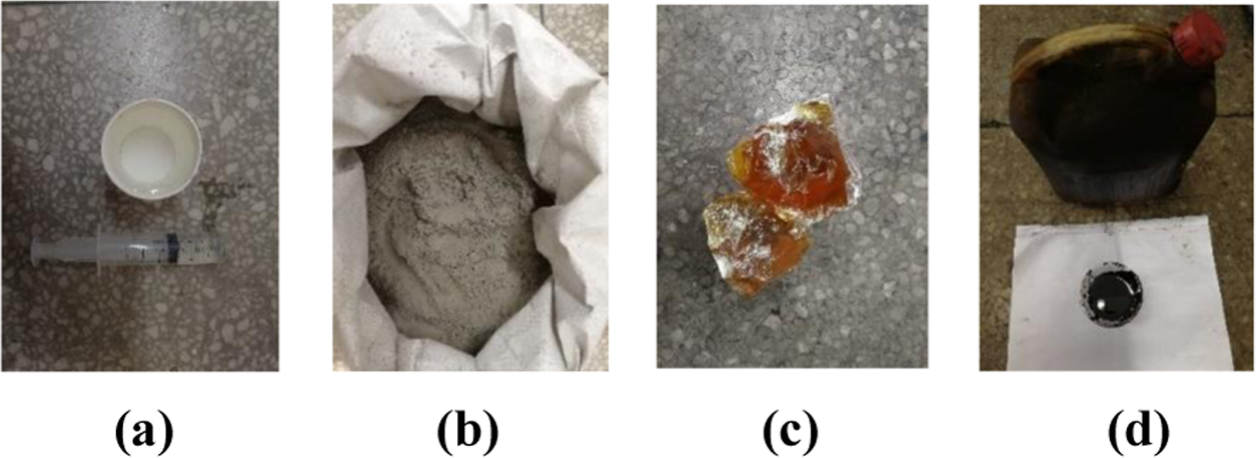
Four kinds of binders used for making the briquette coal samples. (a) Water, (b) cement, (c) rosin, and (d) coal tar.

**Figure 2:**
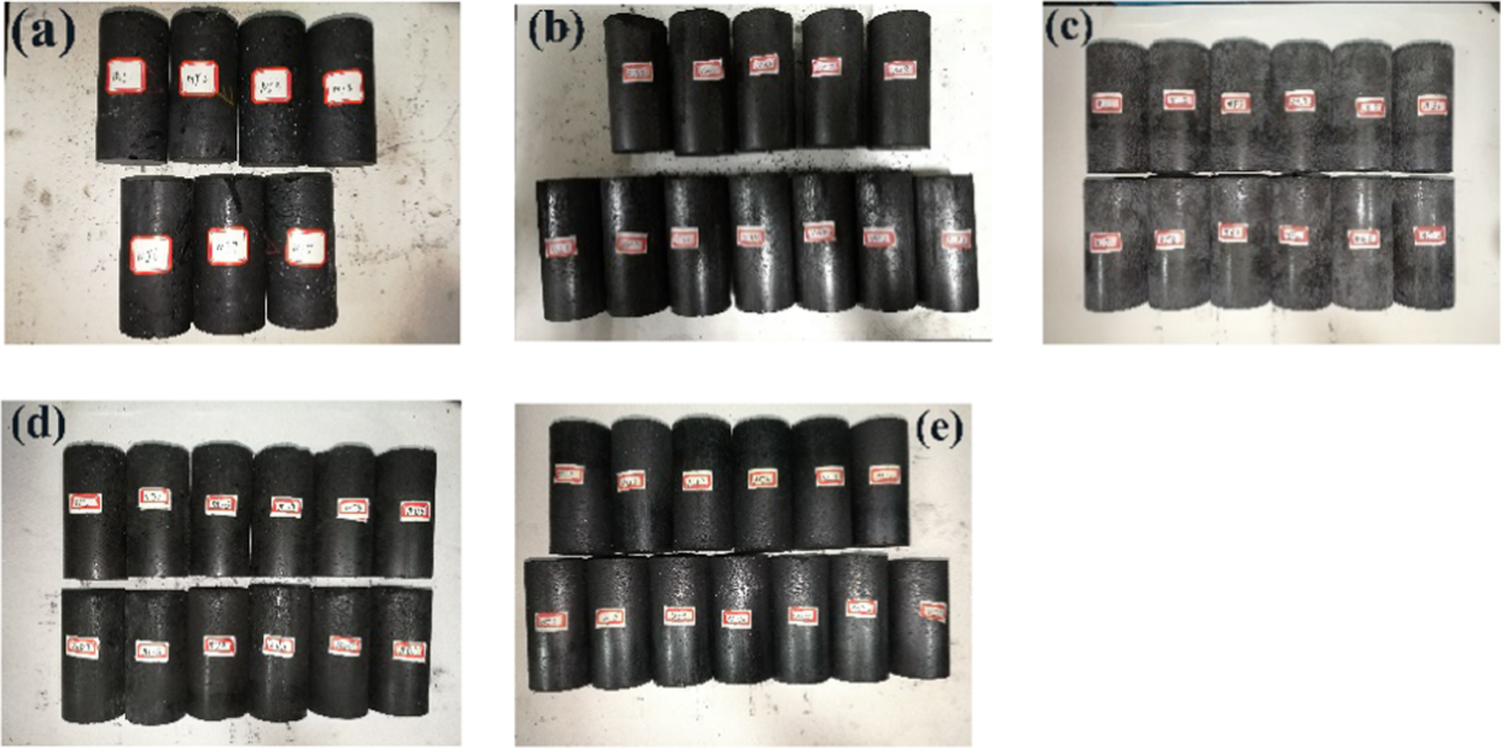
Preparation for the test of raw coal samples (a) and briquette coal samples. (b) MA group, (c) ME group, (d) MD group, and (e) MY group.

Two types of coal samples were prepared in this study. One is a raw coal sample, and the other is a briquette coal sample with four kinds of binders. In the laboratory, a core drill was used to directly drill the raw coal samples from the block coal. Then, a grinder was used to grind and polish; the final coal sample has a standard size of ϕ50 × *h*100 mm. The briquette coal sample was composed of coal powder and different binders. After the raw coal samples core were obtained from the big blocks, the residual blocks were used to crush, and then a standard sieve was used to get three main particle size ranges. The content of pulverized coal particles that make up the briquette coal is equal to 1, and the content of each binder is 7% of the total coal mass (Table 1), which means that the binder quality was unique. The four kinds of binders are water, cement, rosin, and coal tar. As a natural binding agent, water has good affinity and is used as a common additive for making briquette coal samples. Cement has good hydration, hardening, and antiseepage characteristics; rosin has a strong binding force and can be used as a hot-melt adhesive; and coal tar is generally used as a binder for industrial briquette coal samples (Figure 1). The well-proportioned briquette coal material was loaded into the mold, and then the mold was retreated on the press with a pressure of 60 kN for 30 min. The size of the standard briquette coal sample is ϕ50 × *h*100 mm (Figure 2).

**Table 1:** Proportioning Scheme of Briquette Coal Samples

group	coal powder (%)			proportion of binder (%)			
	0–0.3 mm	0.3–1 mm	0.3–1 mm	water	cement	rosin	coal tar
MA	32.5	41.7	25.8	7	0	0	0
ME	32.5	41.7	25.8	8.7	7	0	0
MD	32.5	41.7	25.8	0	0	7	0
MY	32.5	41.7	25.8	0	0	0	7

### 2.2 Experimental Methods

We performed triaxial compression experiments on the raw coal and briquette coal samples in this study. All tests were performed in the Rock Mechanics Laboratory of the Henan Polytechnic University by adopting hydrostatic pressure loading (σ_1_ = σ_3_) and displacement control methods. The specific loading steps are as follows: (1) The setting values of the confining pressure of the raw coal are 2, 5, 10, 15, 20, and 25 MPa. The hydrostatic pressure loading condition is σ_1_ = σ_3_ (Table 2). In the initial stage of loading, force control is used to simultaneously apply axial pressure and confining pressure to reach a predetermined value (at the rate of 0.5 MPa s^–1^). Then, confining pressure was kept at a constant value, and the machine control set into displacement control (at the rate of 0.005 mm s^–1^). Finally, the coal samples are completely damaged due to constant loading. (2) The setting values of the confining pressure of briquette coal samples are 0.2, 0.4, 0.6, 0.8, 1.0, 2.0, 3.0, and 4.0 MPa (Table 2). The control method of the three-axis loading of briquette coal samples is the same as that for the raw coal samples.

**Table 2:** Coal Sample Numbers for the Triaxial Compression Test

coal sample type	binder	number
raw coal		MJ2, MJ3, MJ4, MJ5, MJ6, MJ7
briquette coal	water	MA-6, MA-7, MA-8, MA-9, MA-10
	cement	ME-6, ME-7, ME-8, ME-9, ME-10
	rosin	MD-6, MD-7, MD-8, MD-9, MD-10
	coal tar	MY-8, MY-9, MY-10, MY-11; MY-12

**Figure 3:**
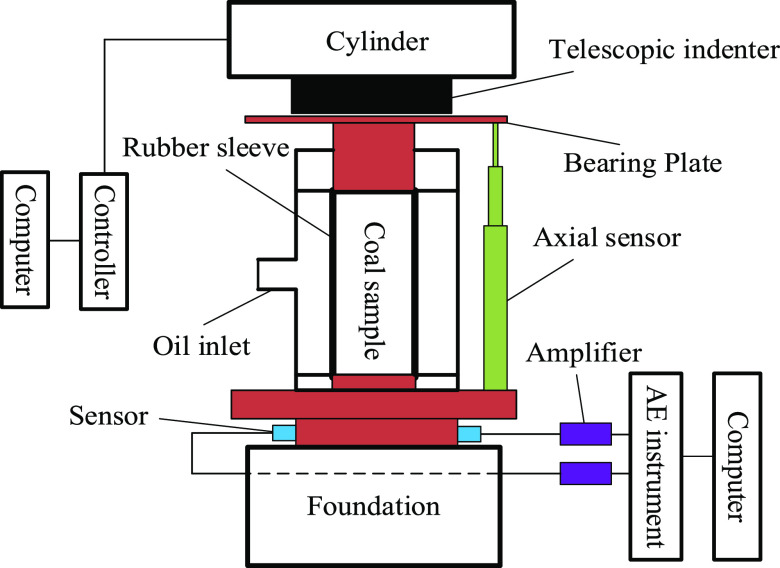
Triaxial compression test system.

**Figure 4:**
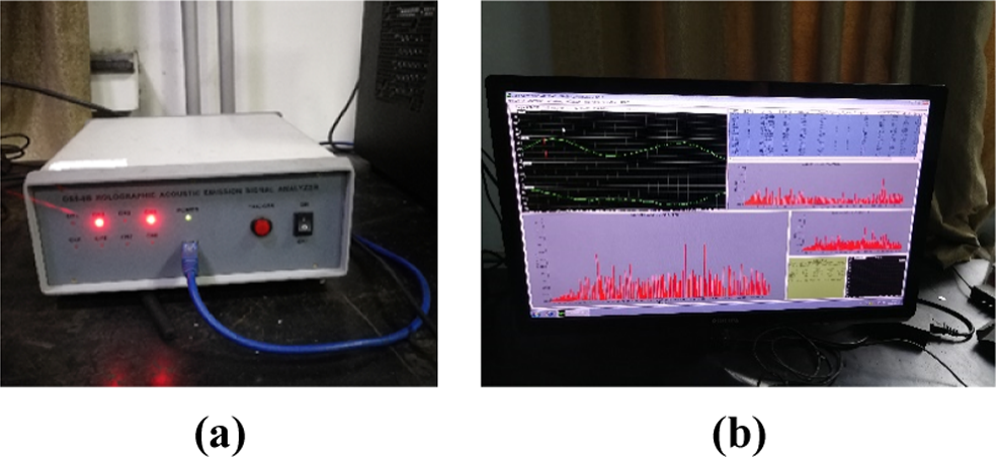
AE monitoring system. (a) AE signal converter and (b) control computer.

In the process of triaxial tests, the AE signals were monitored simultaneously for each coal sample (Figure 3). The two sensors were arranged on the three-axis compression base. Butter was applied to the AE probe to reduce the attenuation in the air of the vibration generated when the coal samples rupture. The sampling frequency was set to 3 MHz, and the sampling threshold was set to 50 dB. The AE signal was recorded by software and can be exported for postprocessing (Figure 4).

## 3 Experimental Results

### 3.1 Deformation of Coal Samples

#### 3.1.1 Influence of Confining Pressure

**Figure 5:**
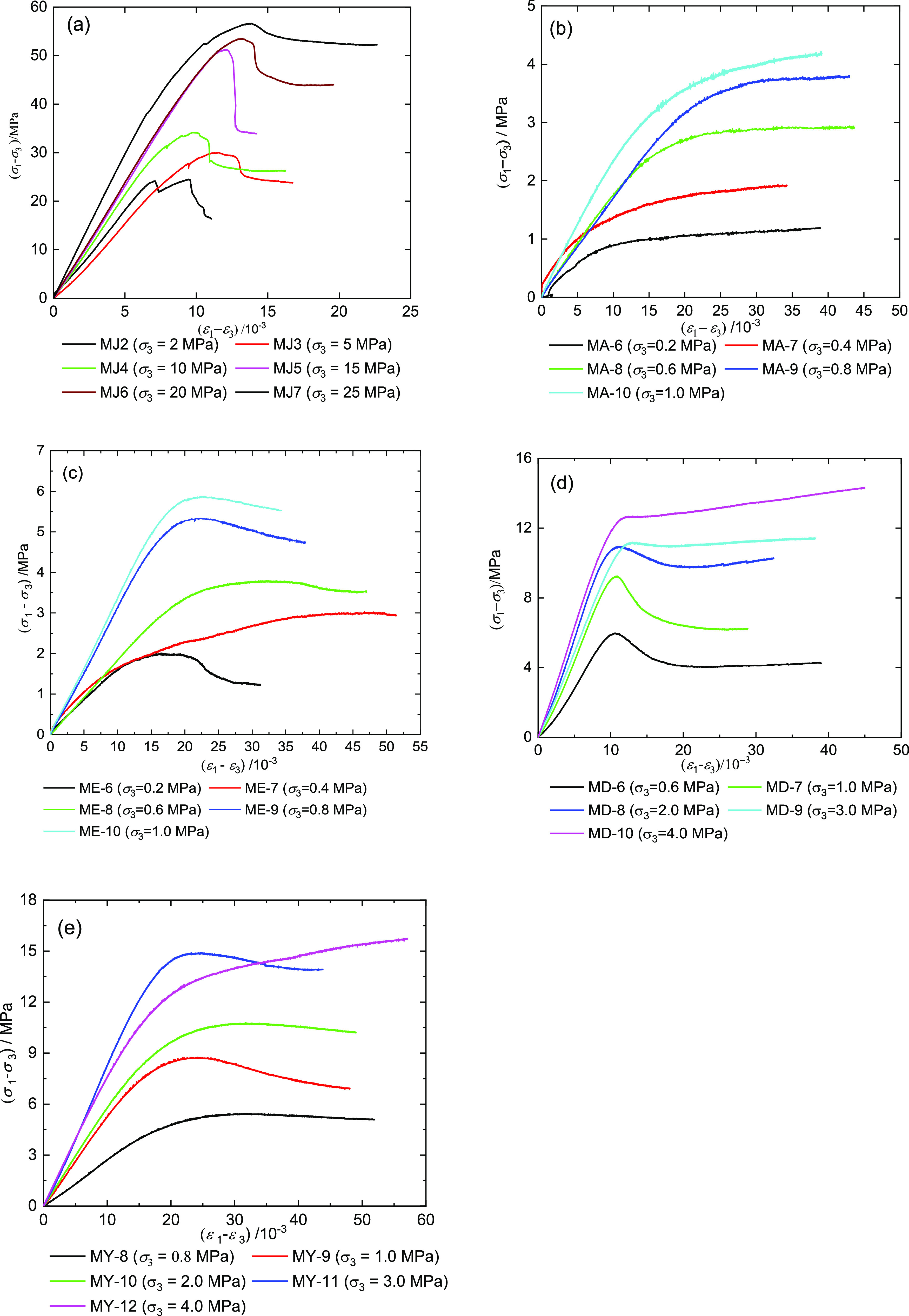
Stress–strain curves of coal samples under the triaxial test. (a) Raw coal samples; (b) MA group, 7% water; (c) ME group, 7% cement; (d) MD group, 7% rosin; and (e) MY group, 7% coal tar.

In the triaxial compression tests, raw coal samples ‘s confining pressure values are 2, 5, 10, 15, 20, and 25 MPa and briquette coal sample’s confining pressure values are 0.2, 0.4, 0.6, 0.8, 1.0, 2.0, 3.0, 4.0, and 5.0 MPa. Figure 5 shows the stress–strain curves of the coal samples recorded during the triaxial compression test, where σ_1_ – σ_3_ is the triaxial compressive strength, MPa, and ε_1_ – ε_3_ is the triaxial compressive strain, 10^–3^.

Figure 5 shows the stress–strain curves of the coal samples recorded during the triaxial compression test. The typical failure process of rock samples always shows four phases: compaction, elastic, yield, and failure stage. Figure 5 shows that the coal samples’ compaction and elastic phases are not obvious border under high confining compression. Therefore, we can easimated that different types of coal samples have different deformation characteristics. The raw coal samples exhibit brittle failure under a low confining pressure (σ_3_ ≤ 20 MPa); when σ_3_ = 25 MPa, the MJ 7 coal sample showed obvious plastic failure characteristics (Figure 5a). For example, under the confining pressure below 20 MPa, the raw coal samples all showed an obvious drop in stress, and the raw coal sample MJ 2 also showed a steep drop of stress and the brittle failure characteristic. Figure 5a shows that before the triaxial compression of the raw coal sample reaches the peak stress, there is no continuous yield stress plateau. As the confining pressure continues to increase, the residual strength of the corresponding raw coal sample also increases and gradually approaches the peak strength. So the assumption was made that when the confining pressure is large enough, the peak strength and residual strength of the coal sample will be equaled. It was found that the peak strain and peak deformation of the coal samples have a little relationship with the confining pressures.

The MA group briquette coal samples mainly show the plastic deformation characteristics (Figure 5b). As the confining pressure increases, the MA group samples show no obvious compaction and elasticity. In the failure stage, the coal samples consistently showed the strain strengthening features. In the failure stage, the MA group also has no obvious stress drops. The appearance of the abovementioned deformation characteristics was indicated by the fact that briquette coal samples have soft and strong plastic characteristics. Therefore, the clear peak strength of each coal sample from the MA group was difficult to find. During the strain strengthening stage, the briquette coal samples continue to undertake the loading strength by relying on the cohesion and internal friction.

We can directly find the peak intensity of the ME group coal samples (Figure 5c). The compaction and elastic stages of ME-9 and ME-10 were relatively close, and the peak strains of the two briquette coal samples were really small.

Except for the ME-7 briquette coal sample, other coal samples of the ME group showed strain-softening characteristics after peak stress. We can estimated that if the waiting time was long enough, the ME-9 briquette coal sample showed strain-softening characteristic. However, the briquette coal samples of the ME group showed plastic failure characteristics. In the failure stages, the ME group coal samples showed softening characteristics. Although the ME-6 coal sample is under a confining pressure of 0.2 MPa, there still appears a significant stress drop after peak stress.

Figure 5d shows that the MD group briquette coal samples exhibited a significant peak intensity when the triaxial loading was completed. We can note that when the confining pressure was beyond 1.0 MPa, the compaction and elastic phases were nearly close. Although the MD group coal samples showed stress drops, the coal samples showed plastic failure. However, the MD-9 and MD-10 briquette coal samples have different degrees of strain-hardening characteristics after peak stress. At the same time, the strain-hardening characteristics of the briquette coal sample of MY-12 can also cause strain strength after the peak stress (Figure 5e). The briquette coal samples of the MY group show failure characteristics that are similar to the ME group. Therefore, the MY-12 coal sample does not participate in the regression of the curve.

**Figure 6:**
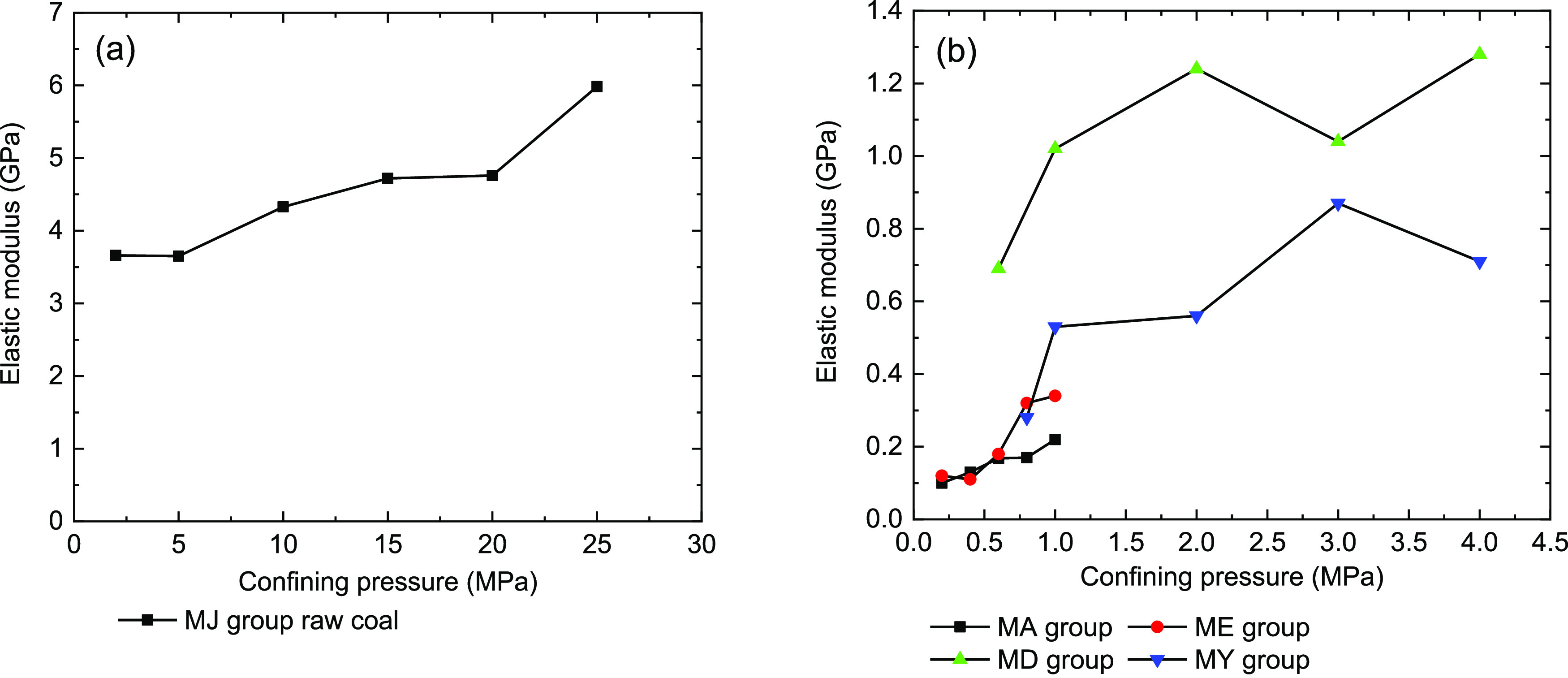
Relationship of confining pressures and the elastic modulus of coal samples under triaxial compression. (a) Raw coal samples and (b) briquette coal samples.

The elastic modulus of the raw and briquette coal samples shows an increasing trend with increased confining pressure (Figure 6a). Therefore, we can suppose that the confining pressure is the reason for the increase in rigidity of the raw coal and briquette coal samples. Under the same confining pressure of 2 MPa, the elastic modulus value of raw coal MJ 2 is 6.54 times that of MY-10 and 2.95 times that of MD-8 of the briquette coal sample. It was found that the low strength and large deformation features of briquette coal samples. Under the same confining pressure conditions (σ_3_ = 1.0 MPa), the MD group briquette coal samples’ elastic modulus was bigger than that of other group’s briquette coal samples. We could find that under the same added quality, the briquette coal sample includes rosin, whose cohesive force was greater than that of water, cement, and coal tar.

**Figure 7:**
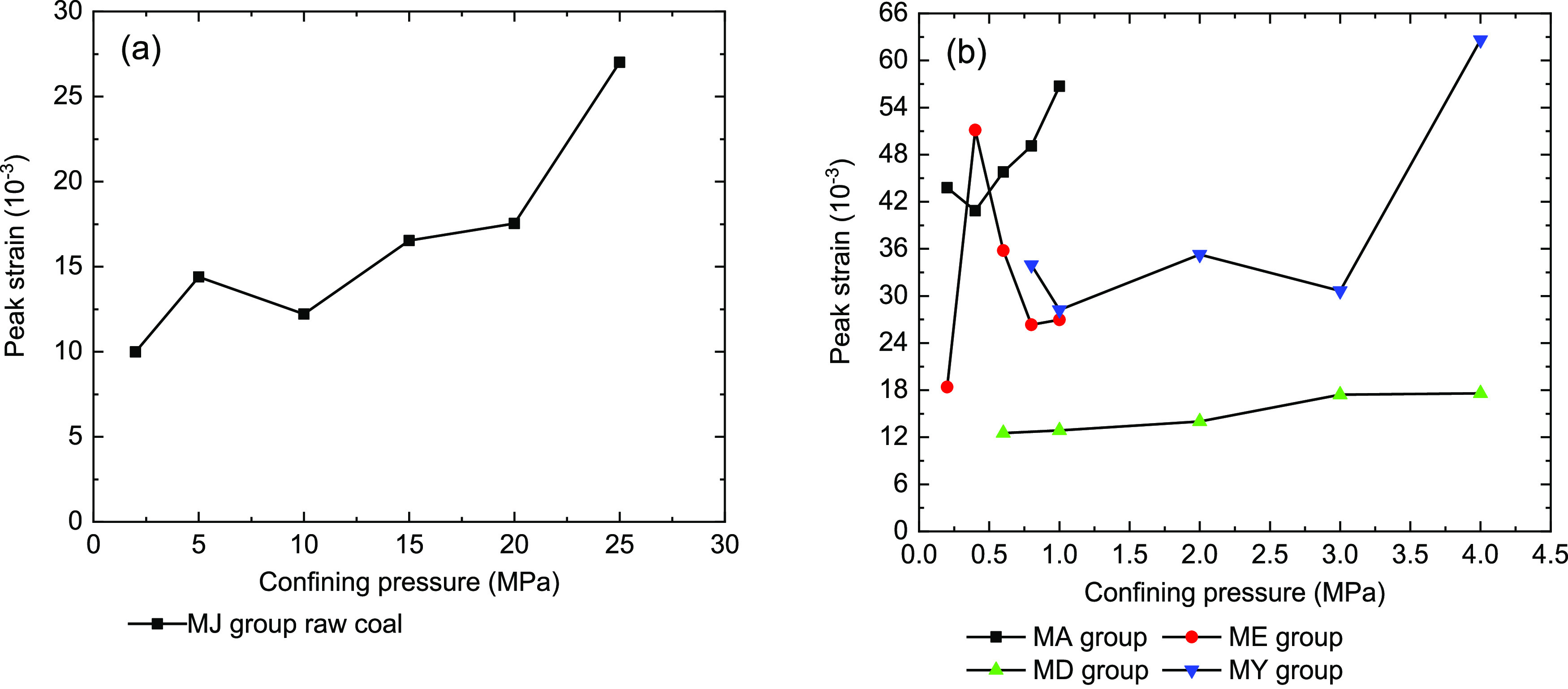
Relationship between the peak strain and confining pressure of coal samples under triaxial compression. (a) Raw coal samples and (b) briquette coal samples.

Most of the coal sample peak strains have a positive relationship with the confining pressure (Figure 7). However, due to the plastic characteristics of briquette coal sample ME-9, the axial deformation of this sample was relatively large. Under the same confining pressure of 2 MPa, the peak strain of the MD-8 and MY-10 briquette coal samples was bigger than that of the MJ-2 raw coal sample. Once again, we found that briquette coal samples have stronger plastic flow features. The group of briquette coal samples showed similar characteristics to the ideal plastic material under triaxial compression tests. Therefore, it can be found that the briquette coal samples had strong plastic characteristics, which led to fluctuating linear characteristics of the peak strain.

#### 3.1.2 Influence of Different Binders

**Figure 8:**
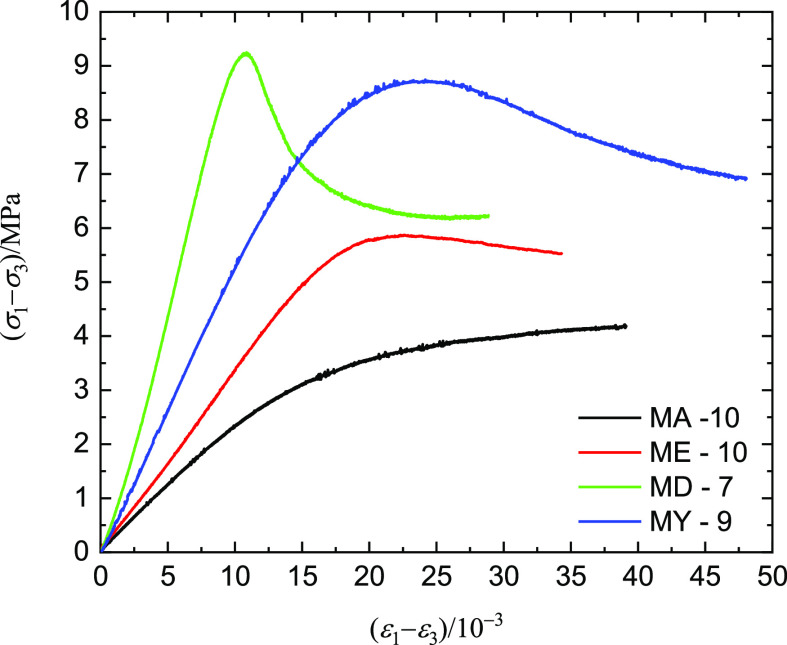
Comparison of stress–strain curves of different binders of briquette coal samples under the triaxial compression test (σ_3_ = 1.0 MPa).

Under the condition that the confining pressure was 1 MPa, the results show that briquette coal samples composed of four binders had different characteristics (Figure 8). Except for the MD-7 briquette coal sample, the other three types of briquette coal samples all showed self-evident plastic failure. After the peak stress process, the ME-10 and MY-9 briquette coal samples show an obvious yield weakening trend. Under equal confining pressures, the peak strains of the MY group were larger than those of the MD group, and the peak strains of the MA group were larger than those of the ME group briquette coal (Figure 7b). The MA-10 briquette coal sample had a 7% water content, and the strength was relatively low. In contrast, the MD-7 briquette coal sample with 7% rosin had a small peak strain because it showed brittle failure characteristics. Under the same conditions of a binder content of 7% and the confining pressures of 1 MPa, we observe that the order of cohesion parameters of the different binders in briquette coal samples was MD group (rosin) > MY group (coal tra) > ME group (cement) > MA group (water).

### 3.2 Coal Samples’ Strength Characteristics

#### 3.2.1 Relationship of Strength and Confining Pressure

The confining pressure has a positive relationship with the strength of each coal sample. As the confining pressure increases, the briquette coal samples also show obvious strain strength characteristics. Table 3 shows the results of raw and briquette coal samples under the triaxial compression test. In Table 3, σ_3_ is the confining pressure, MPa; σ_max_ is the peak intensity, MPa; *E*_T_ is the elastic modulus, GPa; *E*_50_ is the deformation modulus, GPa; *C* is the cohesion, MPa; and φ is the internal friction angle (degree).

**Table 3:** Triaxial Compression Results of Coal Samples

coal sample	σ_3_	σ_max_	*E*_T_	*E*_50_	_*C*_	φ
MJ2	2	26.38	3.66	3.52		
MJ3	5	35.82	3.65	2.72		
MJ4	10	44.64	4.33	4.23	7.19	25.44
MJ5	15	66.84	4.72	5.32		
MJ6	20	73.61	4.76	4.66		
MJ7	25	82.19	5.98	5.94		
MA-6	0.2	1.39	0.1	0.06		
MA-7	0.4	2.36	0.13	0.11		
MA-8	0.6	3.57	0.17	0.15	0.75	41.83
MA-9	0.8	4.63	0.17	0.14		
MA-10	1.0	5.26	0.22	0.11		
ME-6	0.2	2.21	0.12	0.15		
ME-7	0.4	3.45	0.11	0.15		
ME-8	0.6	4.43	0.18	0.18	0.7	45.37
ME-9	0.8	6.16	0.32	0.27		
ME-10	1.0	6.79	0.34	0.28		
MD-6	0.6	6.6	0.69	0.48		
MD-7	1.0	10.3	1.02	0.76		
MD-8	2.0	13.02	1.24	0.93	0.89	27.16
MD-9	3.0	14.27	1.04	0.82		
MD-10	4.0	16.87	1.28	1.04		
MY-8	0.8	6.26	0.28	0.27		
MY-9	1.0	9.79	0.53	0.43		
MY-10	2.0	12.86	0.56	0.58	1.13	37.3
MY-11	3.0	18.03	0.87	0.68		
MY-12	4.0	19.88	0.71	0.74		

However, the internal friction factor of the same group coal samples was constant, the cross-sectional directions of the coal samples were not completely the same, and the cohesive force of each group of coal samples was also different. Therefore, the strength and failure modes of different types of coal samples are different.

**Figure 9:**
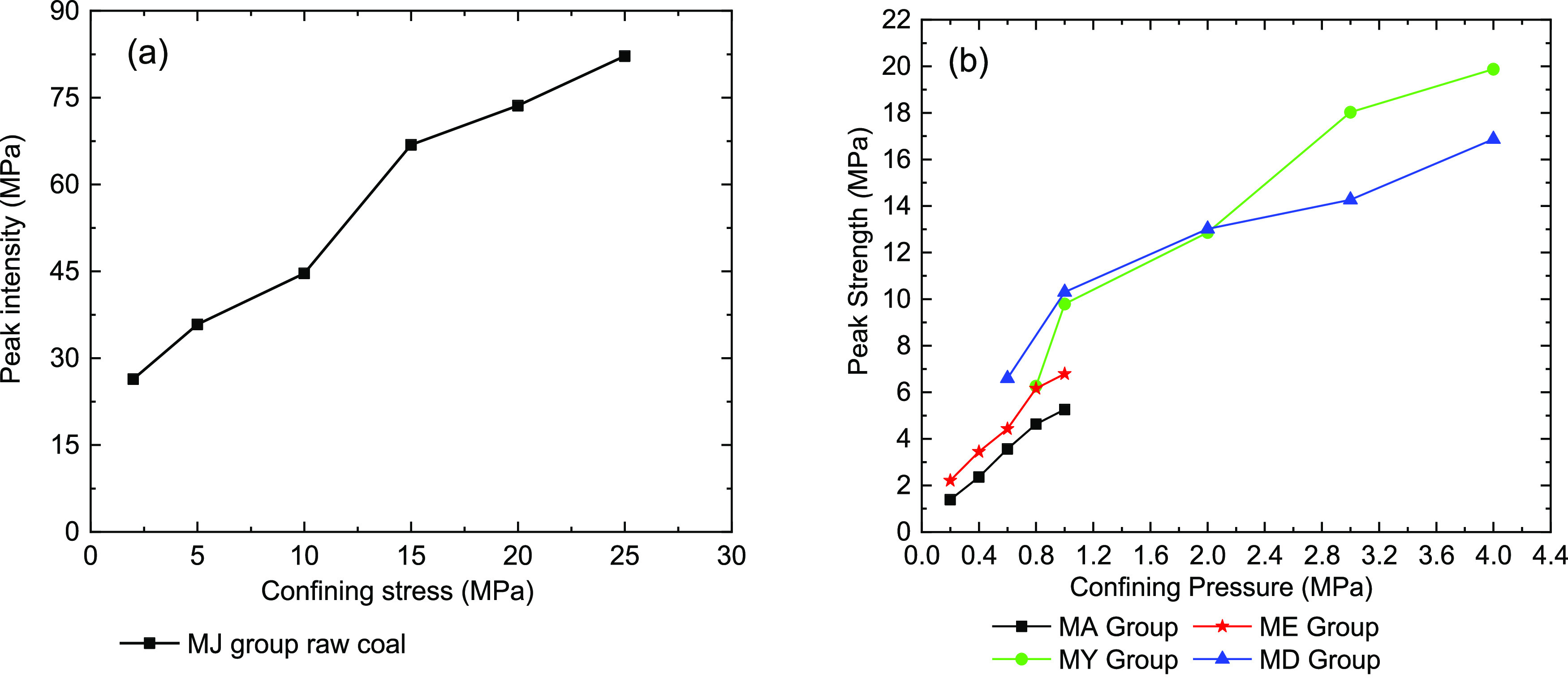
Relationship between the peak strength and confining pressure of coal samples under triaxial compression. (a) Raw coal samples and (b) briquette coal samples.

We also found the relationship between the peak strength and confining pressure of raw and briquette coal samples (Figure 9). For each coal sample group in the experiment, the peak strength increases as the confining pressure constantly increases. The reason for this is that the increase in the strength of the raw and briquette coal samples comes from the increase in the bearing capacity of the material itself. The sliding resistance of each coal body has a positive relationship with the confining pressures, and, in turn, the strength of the raw and briquette coal samples increases.

According to the Mohr–Coulomb strength criterion^40^
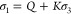
1where σ_1_ is the peak strength, MPa; and *Q*, *K* are the strength parameters of the material. The relationship of the strength parameters with the internal friction angle φ and cohesive force *C* is as follows
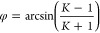
2
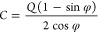
3Regression analysis was carried out on raw and briquette coal samples under different confining pressures of the conventional triaxial test. From the perspective of the confining pressure’s influence on the coefficient *K* value, the briquette coal sample of the MA group is 5.005, ME group is 5.935, MD group is 2.680, and MY group is 4.808 (Table 4). It indicated that the additives in the briquette coal samples have a great impact on the *K* value. The correlation coefficients of raw and briquette coal samples were greater than 0.9, which indicated that the peak strength of the raw and briquette coal samples under triaxial compression has a good correspondence line with the confining pressure. The briquette coal sample of MY-12 did not participate in the regression because the coal sample MY-12 showed no obvious peak strength during triaxial compression and it always showed strain-hardening characteristics after the yielding stage.

**Table 4:** Strength Parameters of the Two Types of Coal Samples

type of coal	coal name	*K*	*Q*	*R*^2^
raw coal	MJ group	2.506	22.75	0.9741
	MA group	5.005	0.439	0.9907
briquette coal sample	ME group	5.935	1.047	0.9858
	MD group	2.680	6.530	0.9180
	MY group	4.808	3.561	0.9539

According to eqs 2 and 3, the cohesion *C* and internal friction angle φ of the two types of coal samples can be calculated, and the results are shown in Table 3. The cohesion force of MJ group raw coal samples was 9.59, 10.27, 8.08, and 6.36 times that of the MA, ME, MD, and MY groups of briquette coal samples. The internal friction angle of the MJ group raw coal samples was 0.61, 0.56, 0.94, and 0.68 times that of the MA, ME, MD, and MY groups of briquette coal samples. So, we can find that compared with raw coal samples, the briquette coal samples have a lower cohesion force and a larger internal friction angle.

The MD group briquette coal samples’ internal angle was very close to the raw coal samples’ internal friction angle. The parameter *Q* in eq 1 can be considered as the uniaxial compression strength of the coal samples under complete shear failure. It is generally regarded as the strength of the material itself. During the uniaxial compression of the coal samples, tensile failure occurs along the axial direction and the actual strength is lower than the material strength. It can be said that parameter *Q* cannot be obtained from a single coal sample, but it needs to be obtained by regression analysis of the triaxial compressive strength of multiple coal samples under different confining pressures.

**Figure 10:**
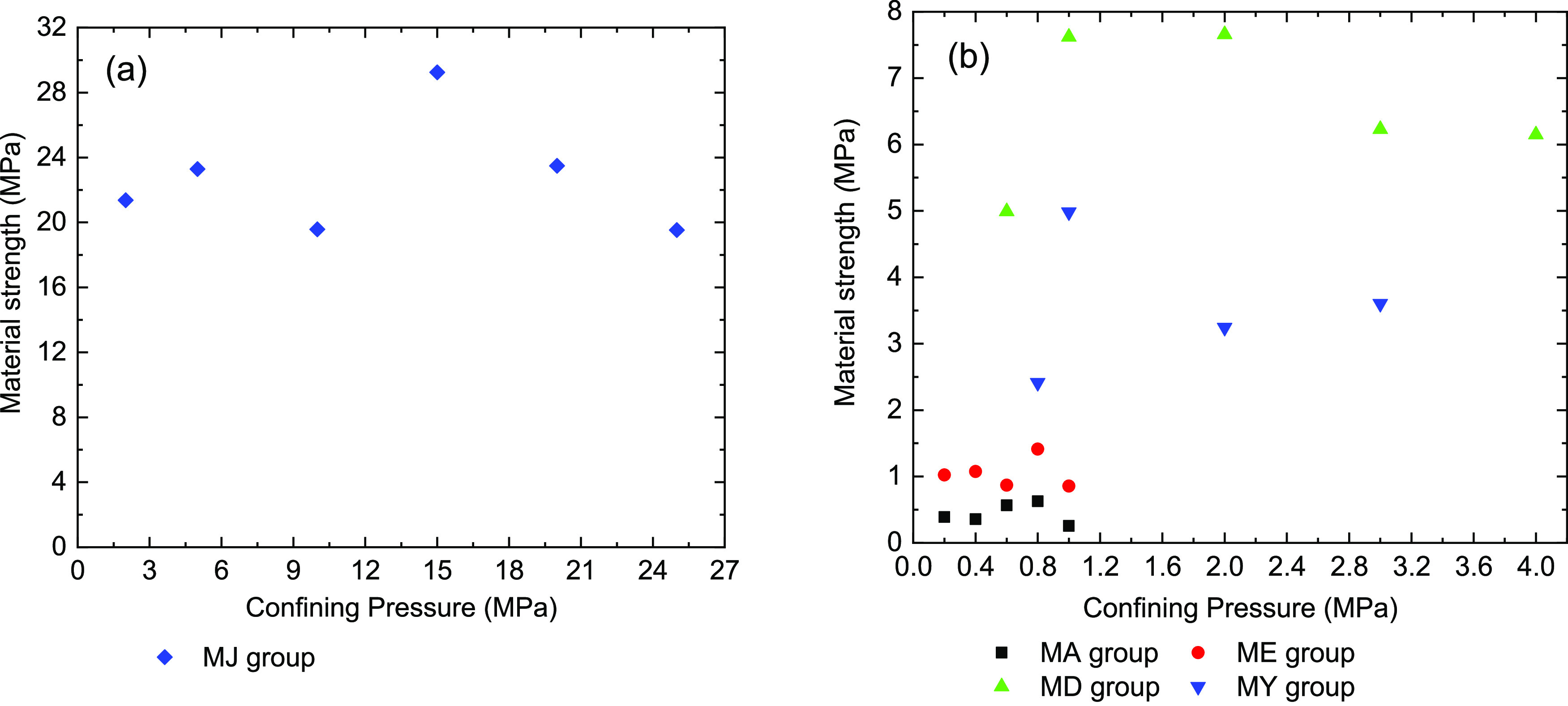
Relationship between the confining pressure and material strengths of coal samples. (a) Raw coal sample and (b) briquette coal sample.

After deducting the effect of confining pressure from the triaxial compressive strength σ_1_ of each coal sample in Figure 5, the material strength *Q* of the coal sample can be obtained.
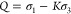
4where the *K* value is the average slope of each straight line shown in Figure 9, corresponding to each coal sample; Table 4 shows the *K* values. Equation 4 can be used to obtain the material strength parameter *Q* of each coal samples. The average value of the strength was equal to the fitting parameter *Q* (in eq 1) value of the confining pressure and strength curves of each group of the coal sample. The material strength for the two types of coal samples is shown in Figure 10. However, the material strength of the raw and briquette coal samples has a certain degree of dispersion. We observe that the material strength of the coal samples changes around the *Q* value. The different binders have a major influence on the strength of the briquette coal samples. Under the same condition, the rosin binders of the MD group coal samples have great strength, and the strength is followed by MY, ME, and MA group briquette coal samples. However, the raw coal samples have bigger material strengths than briquette coal samples.

#### 3.2.2 Destruction Form of Coal Samples

**Figure 11:**
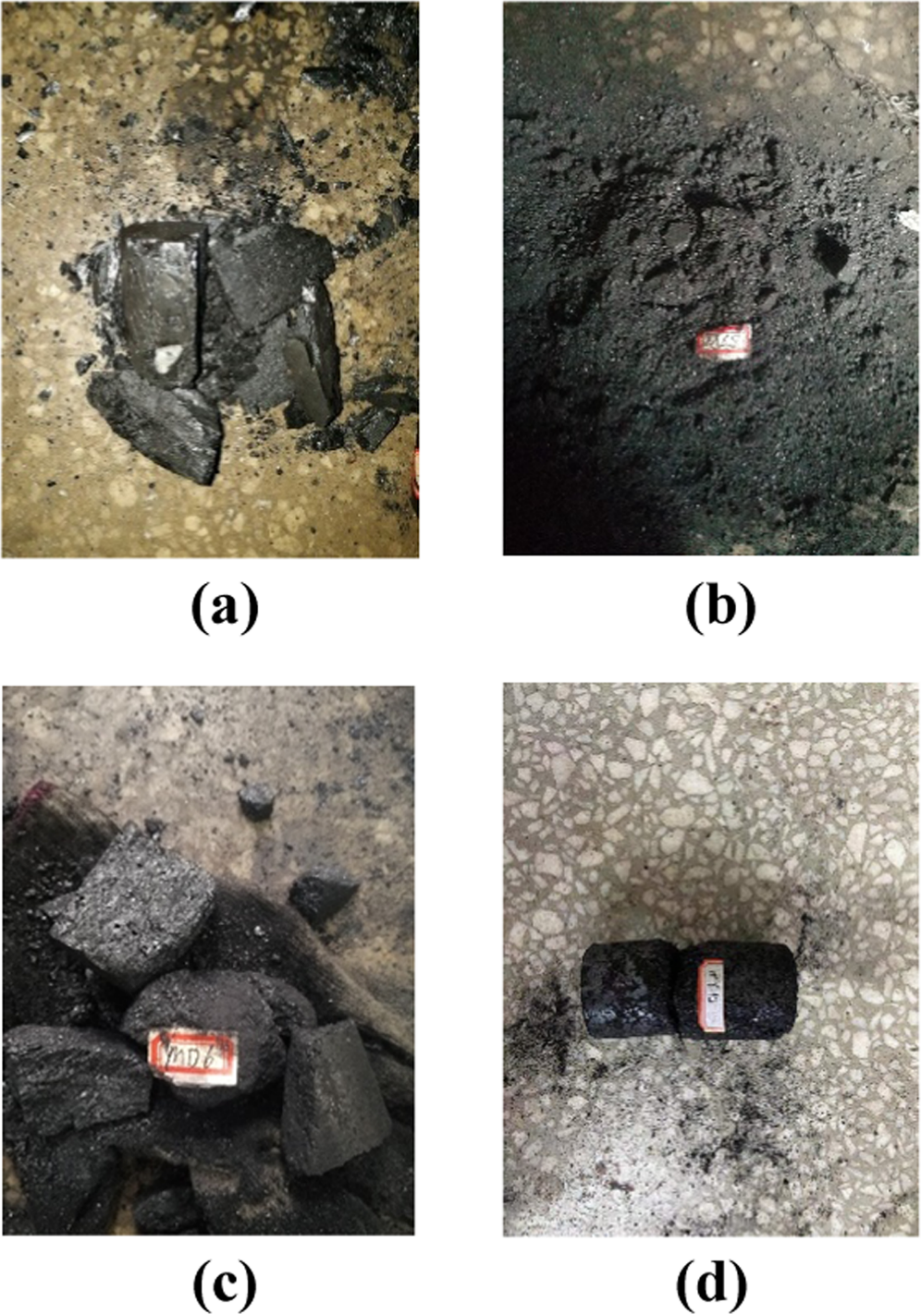
Failure forms of the coal sample under triaxial compression. (a) Raw coal MJ-3 (σ_3_ = 5.0 MPa) and briquette coal samples (b–d): (b) MA-8 (σ_3_ = 0.6 MPa); (c) MD-7 (σ_3_ = 1.0 MPa); and (d) MY-9 (σ_3_ = 1.0 MPa).

**Figure 12:**
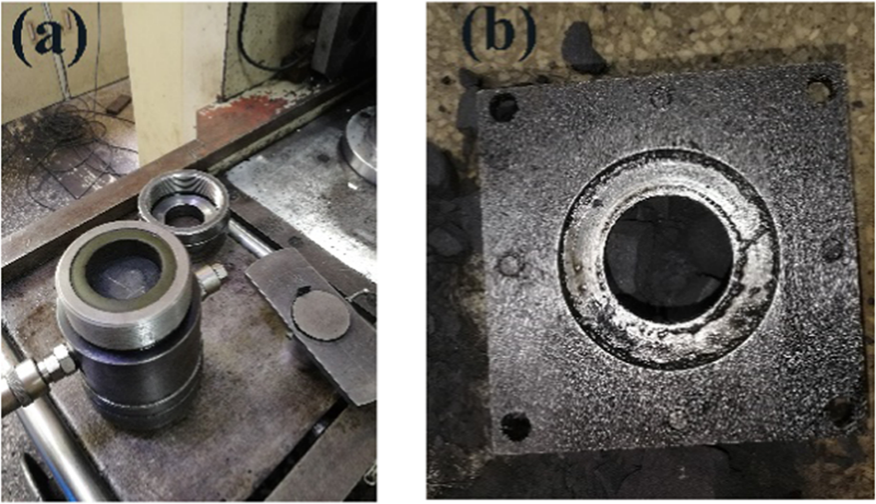
Triaxial pressure cavity and ejection device. (a) Triaxial pressure cavity and (b) mold ejection device.

Data on the morphological damage of the raw and briquette coal samples are given in Figure 11. Our goal here is to show the detailed failure characteristics of each groups’ coal samples. The triaxial compression pressure cavity and ejection device are shown in Figure 12.

When the coal sample triaxial compression was completed, the coal sample was ejected from the device (Figure 12b). However, the coal sample shapes described here were somewhat different from the actual conditions after triaxial compression. Because of its low strength, the briquette coal sample MA-8 produced severe plastic deformation when the confining pressure was 0.6 MPa. Due to the difficulty of the MA-8 briquette coal sample exiting the pressure chamber, it is difficult to distinguish the damaged surface and the type of damage. However, it can be inferred that the failure characteristics of briquette coal sample MA-8 were the shear failure of a single section. In the conventional triaxial compression of the rock samples, the strength of the rock sample’s confining pressure was 0.3445 MPa, which still continuously reduced, and the shear failure form was maintained.^39^ Combine the ref (39) and our experiment, it was found that the MD-7 and MJ-3 briquette coal samples both showed obvious shear failure characteristics, and the failure angles were very close. However, due to the large difference in the essential internal structures of the raw and briquette coal samples, the integrity of the MJ-3 raw coal sample was relatively high, while the MD-7 briquette coal sample was relatively broken. The failure forms of the MY-9 briquette coal sample were not clear, and the coal sample only broke when it was withdrawn from the mold. In general, the failure form of raw and briquette coal samples during triaxial compression was obviously shearing slip failure. Combined with the stress–strain curves (Figure 5), it can be inferred that the load-bearing capacity of the raw and briquette coal samples in the yielding stage can be composed of the cohesion force and internal friction angles of the material.

Combined with Tables 3 and 4, we did comparative research. The internal friction angle of the MD group briquette coal samples is 27.16°, the confining pressure influence coefficient is 2.680, the average material strength is 6.530, and the cohesion is 0.89; the internal friction angle of the MJ raw coal samples is 25.44°, the confining pressure influence coefficient is 2.506, the average material strength is 22.75, and the cohesion is 7.19. Overall, we find that only considering the destruction features, the MD group is similar to the raw coal samples.

### 3.3 AE Parameter Analysis of Coal Samples

AE technology is mainly used to monitor the crack’s changes of instability and destruction of coal rock samples. Under normal circumstances, there are multiple AE parameters to reflect the failure characteristics of coal samples. Three typical AE parameters are selected (amplitude, ring count, and energy) to analyze the failure process of the two types of coal samples. Amplitude commonly reflects the maximum amplitude of the voltage signal released, ring count reflects the rate of recurrence of AE events, and energy reflects the strength of the AE event. It is proportional to the square of the waveform amplitude value of the detected event, and is related to the sampling frequency and environmental noise value of the AE instrument.

**Figure 13:**
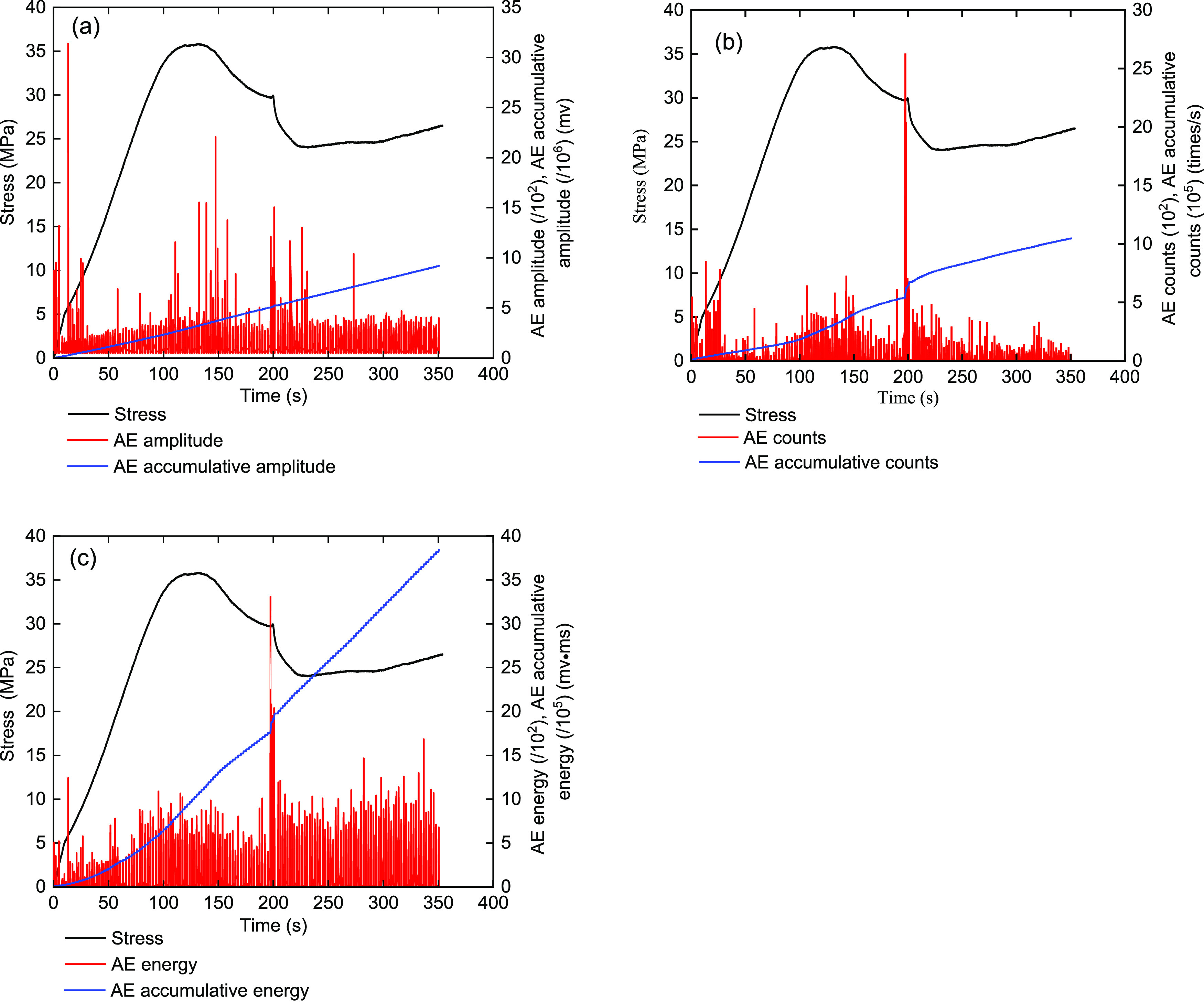
Monitoring results on the AE characteristics of raw coal sample MJ 3 under the triaxial experiment (σ_3_ = 5 MPa). (a) Stress and amplitude, (b) stress and ring counts, and (c) stress and energy.

**Figure 14:**
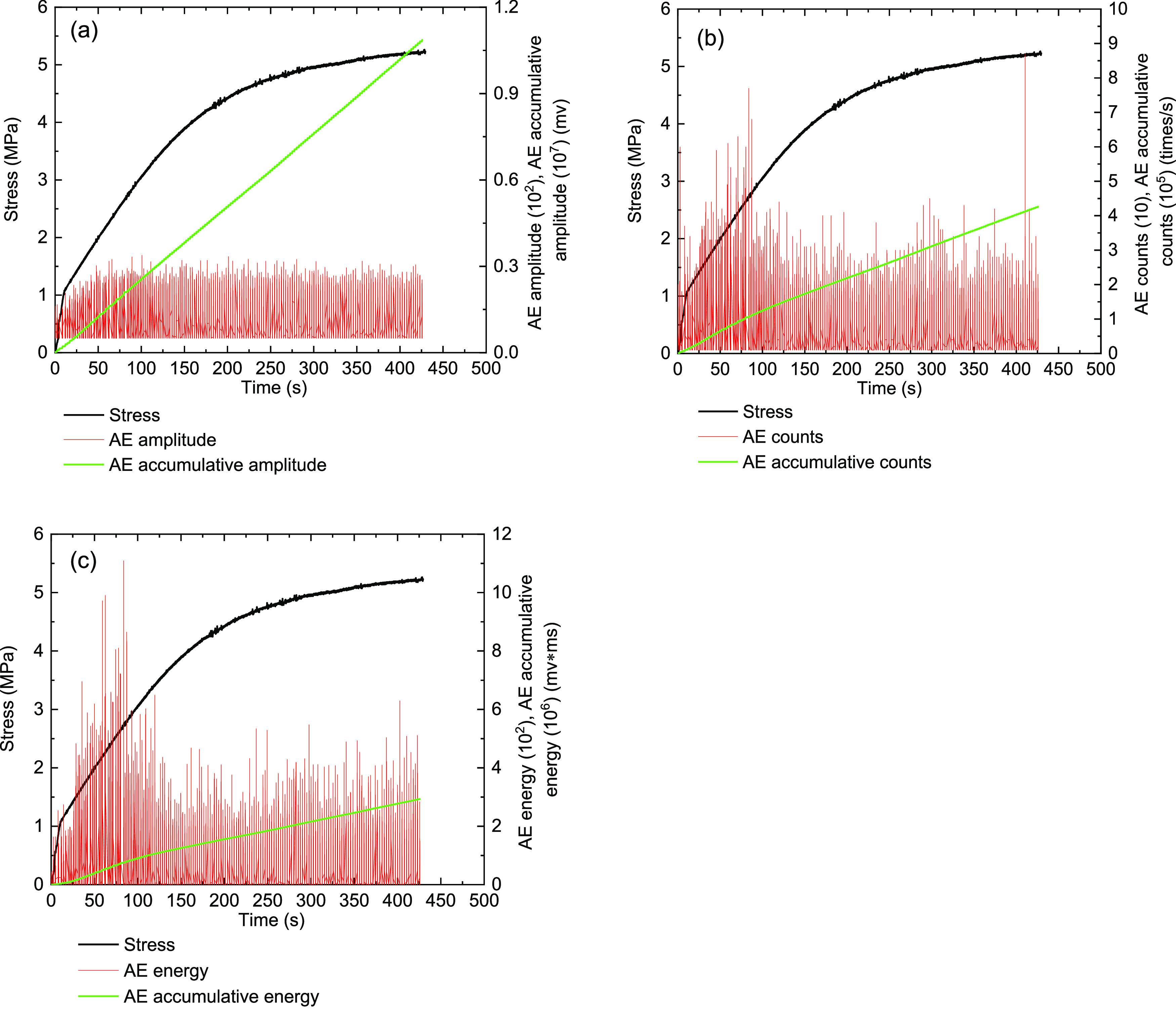
Monitoring results on the AE characteristics of the MA-10 briquette coal sample under the triaxial experiment (σ_3_ = 1 MPa). (a) Stress and amplitude, (b) stress and ring counts, and (c) stress and energy.

**Figure 15:**
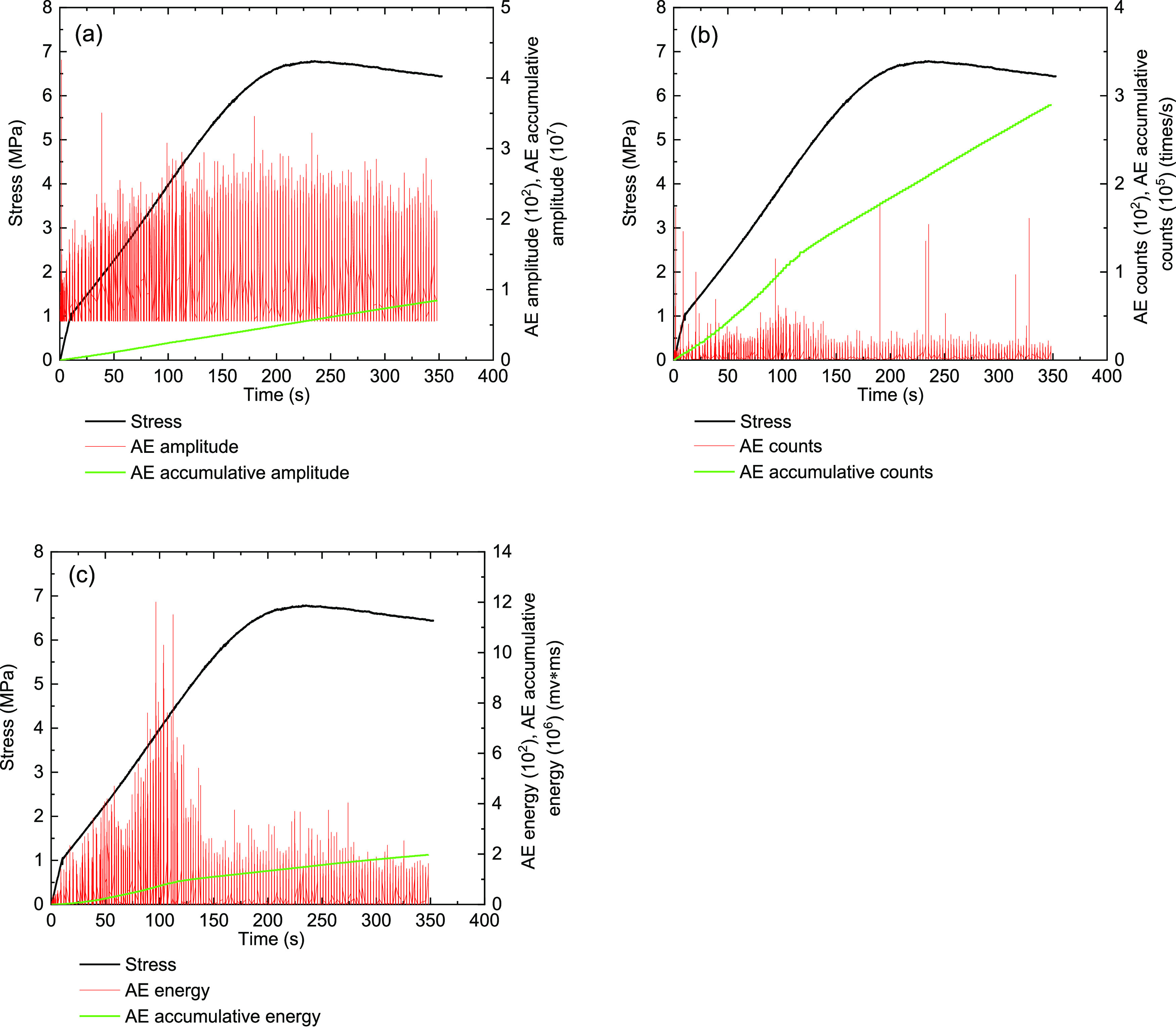
Monitoring results on the AE characteristics of the ME-10 briquette coal sample under the triaxial experiment (σ_3_ = 1 MPa). (a) Stress and amplitude, (b) stress and ring counts, and (c) stress and energy.

**Figure 16:**
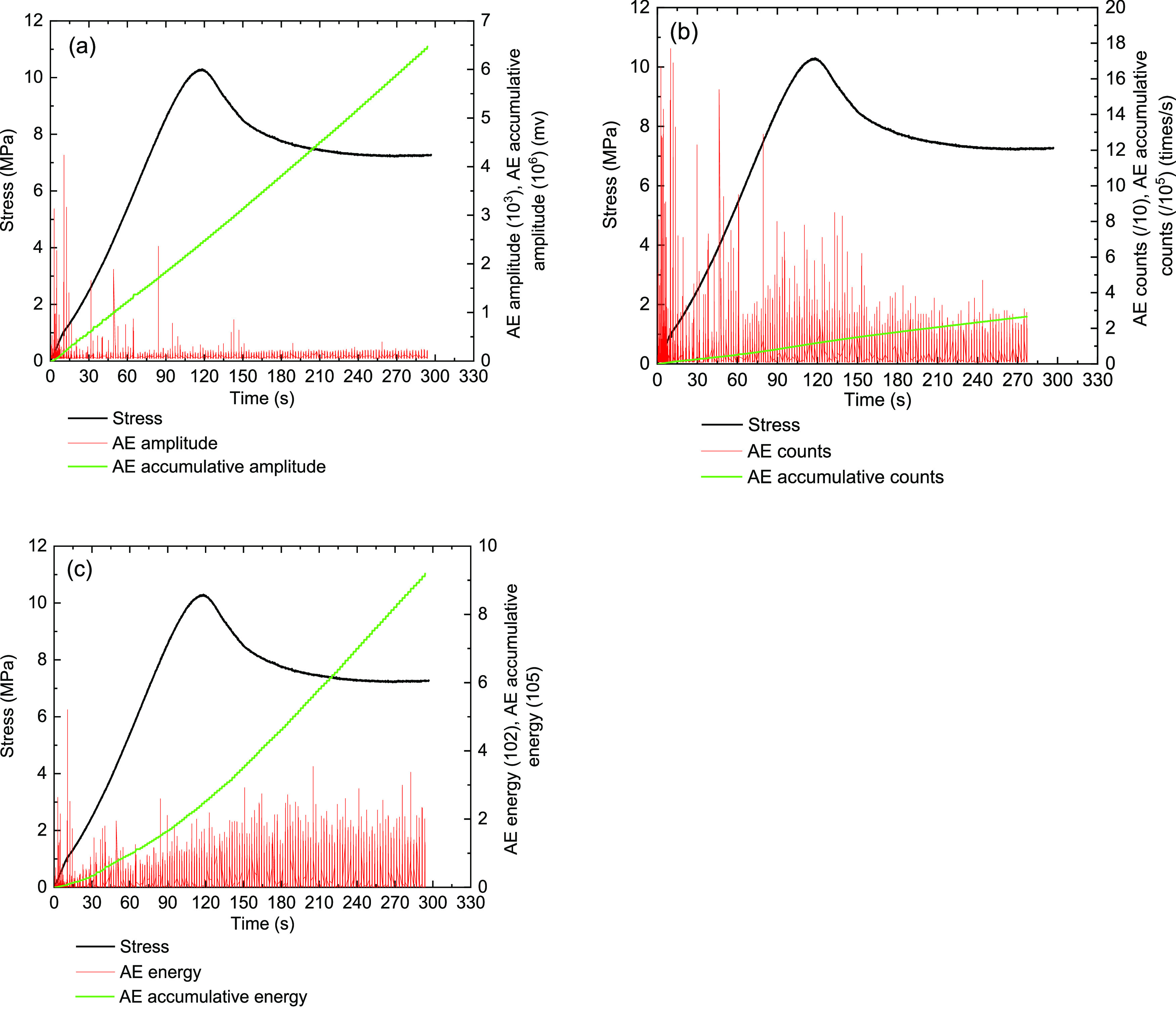
Monitoring results on the AE characteristics of the MD-7 briquette coal sample under the triaxial experiment (σ_3_ = 1 MPa). (a) Stress and amplitude, (b) stress and ring counts, and (c) stress and energy.

**Figure 17:**
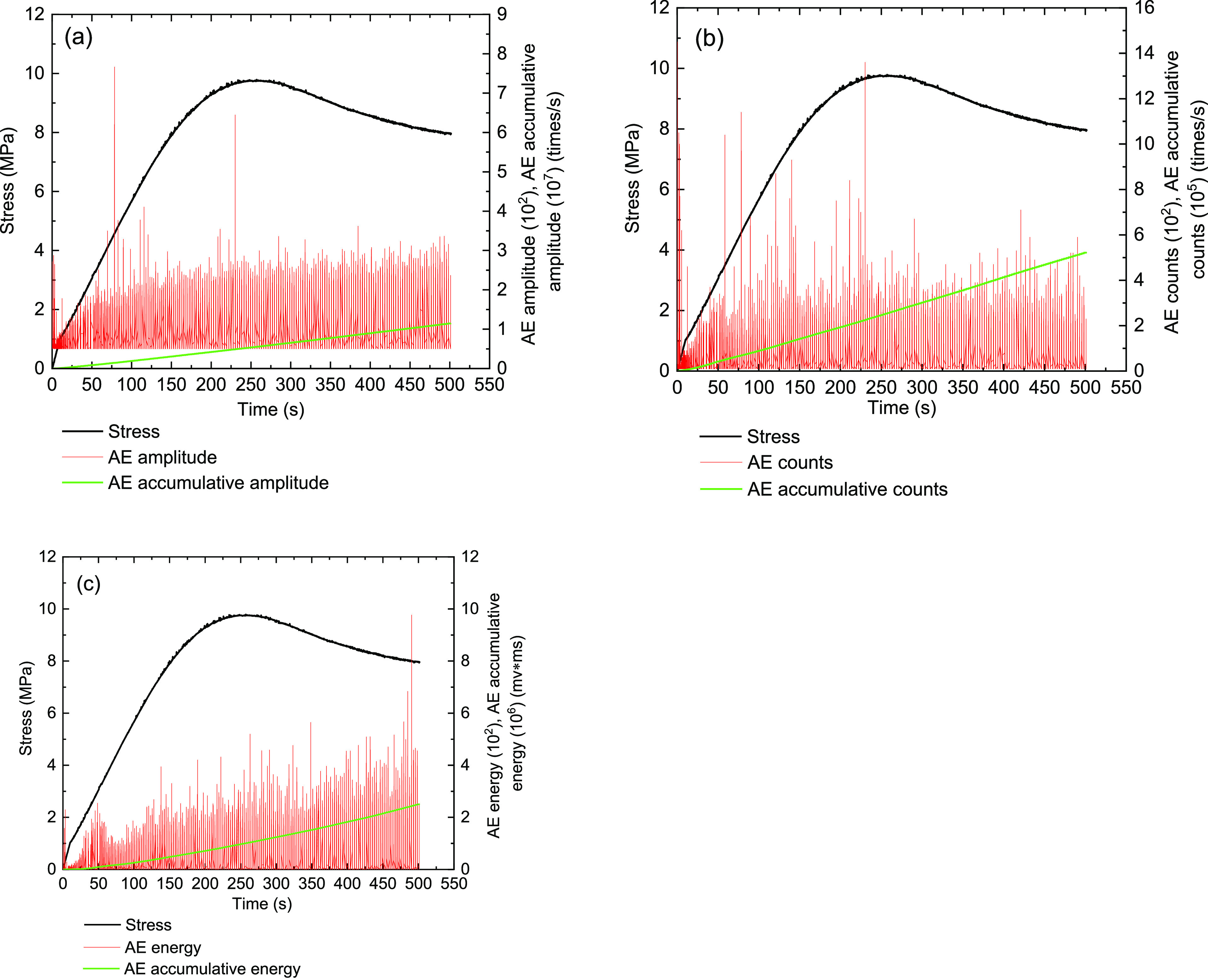
Monitoring results on the AE characteristics of the MY-9 briquette coal sample under the triaxial experiment (σ_3_ = 1 MPa). (a) Stress and amplitude, (b) stress and ring counts, and (c) stress and energy.

Figures 13–17 show the AE events of the raw coal sample MJ 3 under a confining pressure of 5 MPa, and briquette coal samples MA-10, ME-10, MD-7, and MY-9 under a confining pressure of 1 MPa.

The AE characteristics of the raw coal sample of MJ 3 (σ_3_ = 5 MPa) are shown in Figure 13. In the compaction stage, the initial stage of coal sample loading, a certain number of amplitude and energy AE events occurred. It can be observed that the primordial cracks existing in the raw coal samples begin to close. In this process, slippage also occurs between the cracks, and the closure of the internal structural surfaces will also produce a series of AE events. After the coal sample enters the elastic stage, the structure of the coal body begins to bear and deform. Then, the amplitude and ring count of the AE signals begin to appear continuously. The elastic deformation characteristics were so obvious.

When the curves move into the yielding stage, the MJ 3 raw coal sample body begins to undergo initial damage, microcracks begin to appear inside the coal body, and phenomena such as capacity expansion and strain strengthening occur. AE events became active in the yielding stage, and the number of AE events also increased significantly.

After the MJ 3 raw coal sample enters the failure stage, the original cracks in the coal body would expand and gradually form new cracks. At this time, the number of AE events will be more concentrated, and the corresponding peak will be attained simultaneously. Then, the structural surface inside the raw coal body will slip and fail, and it will continue to bear the load depending on the binding force between the particles. AE events in the failure stage were still certainly concentrated, and bigger AE counts and energy are generated during the sudden stress drops. Overall, the AE events had a good relationship with the destruction process of the raw coal sample.

Due to the good homogeneity of the briquette coal sample, the compaction and elasticity stages of this type of coal sample during triaxial compression were not clearly distinguished. The AE signals of briquette coal sample MA-10 showed an increasing trend under the compaction and elastic stages. Then, the ring counts and energy showed a concentrated peak. When the briquette coal sample MA-10 enters the yielding stage, the amplitude continues to stabilize, and the ring count and energy decrease. Therefore, it can be considered that the briquette coal sample is cracked before and slipped along the structural surface.

When the briquette coal sample MA-10 of stress−strain curves into a failure stage, the stress transforms to strain-hardening. Then, the ring count and energy show a peak, whose values were below the peak intensity of the compaction and elastic stages. The destruction stage can be estimated for the briquette coal sample depending on the cohesion force, and the internal friction continues to bear the load.

The AE features of briquette coal sample ME-10 were similar to those of coal sample MA-10, as shown in Figure 15. During the elasticity and compaction stages of coal sample ME-10, the AE signals begin to increase gradually. In the elastic phase, the AE signals of coal sample ME-10 were relatively concentrated, and the energy values reached the maximum. As the loading continues, the briquette coal sample ME-10 enters the yielding stage, the amplitude continues to stabilize, and the ring count and energy values decrease. When the briquette coal sample ME-10 enters the failure stage, the stress begins to decrease, and the strain-softening characteristic appears. During the failure stage, the ring count showed a little peak, the amplitude appeared concentrated and steady, and the energy showsed a decrease with the drop of stress. It can be considered that briquette coal samples of MA-10 and ME-10 have low strength and obvious strong plastic features. Overall, we found that the addition of 7% cement into coal samples cannot obviously change the strength of briquette coal samples.

The triaxial tests of briquette coal samples MD-7 and MY-9 are shown in Figures 16 and 17. In the initial stage of loading, they were in the rising process of confining pressure preloading. However, the briquette coal samples MD-7 and MY-9 showed obvious AE events in the process of the experiments. When the briquette coal sample comes into the compression and elastic stages, the coal samples of AE events have a proportional relationship with time. It can be understood that internal cracks have appeared in the coal samples during the compaction and elasticity stages, and then the cracks continue to develop. The AE ring counts of briquette coal samples were concentrated in the abovementioned stages. In the yielding stage, the briquette coal sample MY-9 has an obvious a yield stress platform, and AE events bigger than the MD-7 briquette coal sample. The coal sample MD-7 triaxial compression process has no obvious yield stress platform, and the brittle deformation characteristic can be seen in Figure 16. Results from Figures 16 and 17 indicated that the two coal samples of AE energy have a positive correlation with time.

When we analyzed the AE events in this study, the preloading process of each group of coal samples was not considered. The AE amplitude, ring count, and energy of MJ-3 raw coal sample was mainly concentrated in the yield and failure stages. In the failure stage, when the stress–time curve drops, the stress drops sharply, and the AE signals at the stress drop point have a corresponding peak. After the raw coal sample of MJ-3 reached the peak stress, a short yield stress plateau appears. At the stage of the yield stress platform, the AE signals begin to develop intensively. The AE events can well reflect the whole failure process of the coal samples. For each group of coal samples, we had selected a representative coal sample; the AE signal parameters are shown in Table 5, where σ_3_ is the confining pressure, MPa; ∑*A* is the AE cumulative amplitude, mv; ∑*N* is the cumulative count, times/s; and ∑*E* is the cumulative energy, mv·ms.

**Table 5:** AE Test Results of the Coal Sample Triaxial Experiment

coal sample type	number	σ_3_	∑*A* (10^7)^	∑*N* (10^5^)	∑*E* (10^6^)
briquette coal sample	MA-10	1	1.09	4.27	2.94
	ME-10	1	0.84	2.90	1.97
	MD-7	1	0.647	2.67	0.92
	MY-9	1	1.15	5.22	2.51
raw coal sample	MJ-3	5	0.922	1.049	3.847

From Tables 4 and 5, it can be found that when σ_3_ = 1 MPa, the four groups of briquette coal samples of AE cumulative amplitude, ring count, and energy have no inevitable linear relationship with the material strengths. The briquette coal sample MD-7 has the strongest material strength, but the AE accumulated energy value is the smallest. The reason for the abovementioned characteristics is that the briquette coal sample has strong plastic characteristics, and it was hence so difficult to cause a stress drop under triaxial experiments. After the peak stress, raw and briquette coal samples can continue to be loaded depending on their own friction and cohesion. Finally, the AE signals can be used to express the failure characteristics of each stage using the triaxial tests of all coal samples.

## 4 Conclusions

The conventional triaxial compression methods were used to measure raw coal and briquette coal samples. The confining pressure and different binders influencing the coal samples’ strength were studied. Additionally, the material strength and AE signals of raw coal and briquette coal samples were analyzed. The main conclusions are as follows:

(1) The raw and briquette coal samples’ peak strength increases as the confining pressure constantly increases under triaxial compression tests. Four types of binders were added into the briquette coal samples; we found that 7% content of rosin of MD group’s briquette coal samples has the biggest peak strength and material strength, and 7% content water of the MA group has minimum strength values. So, we can estimate that the 7% rosin briquette coal samples are most similar to the raw coal samples.
(2) The AE signals can express each stage of all coal samples’ failure characteristics under the triaxial tests. The typical AE signals show that the MA-10 briquette coal samples have bigger AE event values, but the AE accumulated energy value of briquette coal sample MD-7 is the smallest. We can estimate that the MA-10 briquette coal samples show mainly plastic failure, and the MD-7 briquette coal sample shows brittle failure. The AE evolution characteristics of the MD-7 briquette coal sample are similar to those of the raw coal sample.
